# Metallothionein 2 (*SaMT2*) from *Sedum alfredii* Hance Confers Increased Cd Tolerance and Accumulation in Yeast and Tobacco

**DOI:** 10.1371/journal.pone.0102750

**Published:** 2014-07-17

**Authors:** Jie Zhang, Min Zhang, Shengke Tian, Lingli Lu, M. J. I. Shohag, Xiaoe Yang

**Affiliations:** MOE Key Laboratory of Environment Remediation and Ecosystem Health, College of Environmental and Resource Sciences, Zhejiang University, Hangzhou, China; Universidade Federal de Vicosa, Brazil

## Abstract

Metallothioneins are cysteine-rich metal-binding proteins. In the present study, *SaMT2*, a type 2 metallothionein gene, was isolated from Cd/Zn co-hyperaccumulator *Sedum alfredii* Hance. *SaMT2* encodes a putative peptide of 79 amino acid residues including two cysteine-rich domains. The transcript level of SaMT2 was higher in shoots than in roots of *S. alfredii*, and was significantly induced by Cd and Zn treatments. Yeast expression assay showed *SaMT2* significantly enhanced Cd tolerance and accumulation in yeast. Ectopic expression of *SaMT2* in tobacco enhanced Cd and Zn tolerance and accumulation in both shoots and roots of the transgenic plants. The transgenic plants had higher antioxidant enzyme activities and accumulated less H_2_O_2_ than wild-type plants under Cd and Zn treatment. Thus, *SaMT2* could significantly enhance Cd and Zn tolerance and accumulation in transgenic tobacco plants by chelating metals and improving antioxidant system.

## Introduction

Heavy metals are known to cause toxic effects and inhibition of plant growth. However, rare plant species, which can accumulate and tolerate extremely high concentrations of heavy metals in their shoots without toxicity effects, have been defined as “hyperaccumulators” [1]. The elucidation of the mechanisms underlying metal hyperaccumulation may enable the phytoremediation of metal-contaminated soils and the biofortification of trace elements in food crops [2]–[4].

Higher plants have evolved various defense mechanisms to detoxify excess metals. These mechanisms contain compartmentalization in inactive tissues, chelation by metal ligands and detoxification by antioxidants [5]. Metal chelators such as organic acids, amino acids, phytochelatins and metallothioneins play important roles in metal detoxification [6]. Metallothioneins (MTs) are low-molecular-mass, cysteine-rich proteins which are broadly distributed in microorganisms, plants and animals [7]. Plant MTs can be divided into four subfamilies based on the distribution of cysteine residues in their amino- and carboxyl-terminal regions [8]. Several MT genes have been isolated and characterized from plants. There are some evidence indicating that plant MTs are involved in metal homeostasis, detoxification and reactive oxygen species (ROS) scavenging [8]–[11].

Hyperaccumulating ecotype (HE) of *Sedum alfredii* Hance is a Zn/Cd hyperaccumulator discovered from an old Pb/Zn mining area of China [12], [13]. It can accumulate up to 9000 µg g^−1^ Cd and 29000 µg g^−1^ Zn in its shoots without toxicity symptoms [12], [13]. This large amount of metals in plant cells needs a powerful detoxification system to protect plants from the deleterious effects of the metals. Earlier studies have demonstrated that the hyperaccumulating ecotype of *Sedum alfredii* has a more effective antioxidant enzyme system than non-hyperaccumulating ecotype (NHE) [14], [15]. However, the mechanism of hypertolerance of metals in this species has not been fully understood. In the present study, a metallothionein gene from hyperaccumulating ecotype of *Sedum alfredii* Hance, named *SaMT2*, was isolated and cloned The expression pattern of this gene was studied by Real Time-PCR. To analysis the function of *SaMT2*, its full length cDNA was cloned and expressed in yeast and tobacco. The transgenic yeast and tobacco plants were analyzed to evaluate whether *SaMT2* protein played a role in Cd or Zn tolerance and accumulation.

## Materials and Methods

### Ethics statement

These field studies did not involve any protected species. No specific permits were required for the collection of samples in the study location.

### Plant growth

The hyperaccumulating ecotype of *S. alfredii* Hance was collected from an old Pb/Zn mining site in Zhejiang Province, P. R. China. Plants were grown in non-polluted soils for several generations to minimize internal metal concentrations. Similar size shoot branches were cut and cultured hydroponically. After two weeks, rooted seedlings were then subjected to 4 days exposure of one-fourth, half and full strength nutrient solutions containing 2 mM Ca(NO_3_)_2_, 0.7 mM K_2_SO_4_, 0.1 mM KH_2_PO_4_, 0.1 mM KCl, 0.5 mM MgSO_4_, 10 µM H_3_BO_3_, 0.5 µM MnSO_4_, 5 µM ZnSO_4_, 0.2 µM CuSO_4_, 0.01 µM (NH_4_)_6_·Mo_7_O_24_, and 20 µM Fe-EDTA. Nutrient solution pH was adjusted daily to 5.8 with 0.1 M NaOH or HCl. Plants were grown under glasshouse conditions with natural light (day/night of 16/8 h), day/night temperature of 26/20°C and day/night humidity of 70/85%. The nutrient solution was aerated continuously and renewed every 3 d. To compare the expression of *SaMT2*, the precultured seedlings were treated with 100 µM CdCl_2_ and 500 µM ZnSO_4_ for 8 days.

### Cloning of *SaMT2* cDNA and sequence analysis

The cDNA fragment of *SaMT2* was isolated from RNA-Seq data of *Sedum alfredii* Hance (Gao et al. 2013). The full length of *SaMT2* was isolated using 3′ and 5′ RACE methods as described by the supplier (Smart RACE cDNA amplification kit; Clontech Laboratories, Inc. CA, USA). PCR was performed with the following primers: 5′-CTGGGCGTGGCTCCGAAGCAAGTGTA-3′ for 3′ RACE and 5′-CGCAACCACAGTTTCCACCACAGCA-3′ for 5′ RACE. Alignment of *SaMT2* was performed by ClustalW on the internet (http://clustalw.ddbj.nig.ac.jp/). The phylogenetic tree was constructed using the neighbor-joining algorithm by MEGA 5 software (released from http://www.megasoftware.net/) after ClustalW alignment with 1000 bootstrap trials.

### Real time RT-PCR analysis

The total RNA was extracted from various tissues by RNAiso plus (Takara Bio, Inc. Shiga, Japan), and then converted to cDNA using Primescript™ RT regent kit with gDNA eraser (Takara Bio, Inc. Shiga, Japan). Expression of the *SaMT2* was determined by quantitative RT-PCR with the SYBR Green I reagent (SYBR Premix Ex Taq II; Takara Bio, Inc. Shiga, Japan) on an Eppendorf Mastercycler Epgradient Realplex^2^ (Eppendorf AG, Hamburg, Germany). A portion (10 ng) of cDNA was used for the template. The primers used for *SaMT2* were forward 5′-CTGTGGTTGCGGATCTGCTT-3′ and reverse 5′- TCCATTCTCCGACACCATCT-3′. To generate standard curves for the absolute quantification for *SaMT2* copy number, a series of dilutions (from 1×10^−1^ to 1×10^−6 ^ng) of plasmids were made and then subjected to real-time PCR.

### Plasmids construction

To express *SaMT2* in *Saccharomyces cerevisiae*, the full ORF of *SaMT2* was amplified from the cDNA of *S. alfredii* using primers: 5′-AGCTCGAGATGTCTTGCTGTGGTGGA-3′ contains an XhoI site and 5′-GAGGATCCTCATTTGCAAGTGCAGGG-3′ contains a BamHI site. The PCR products were then cloned into a pEASY Blunt simple vector (Transgen, Beijing, China) and its sequence confirmed. This vector was double digested with XhoI and BamHI, and the obtained fragment was cloned into pDR195 between XhoI and BamHI sites.

To construct the plant overexpression vector, the full ORF of *SaMT2* was amplified using primers: 5′-AAAGATCTGATGTCTTGCTGTGGTGGA-3′ and 5′-AAGGTGACCTCATTTGCAAGTGCAGG-3′, which contained BglII and BstpI restriction sites, respectively. The obtained fragment was restricted with BglII and BstpI, and then cloned into the BglII and BstpI sites of pCAMBIA 1302 vector and its sequence was confirmed.

### Yeast complementation assay

The *S. cerevisiae* strains BY4741 (wild type, *MATα*; *his2Δ0; met15Δ0; ura3Δ0), Δycf1 (MATa; his3Δ1; leu2Δ0; lys2Δ0; ura3Δ0; YDR135c::kanMX4*) and *Δzrc1 (MATα; his3Δ1; leu2Δ0; met15Δ0; ura3Δ0; YMR243c::kanMX4)* mutants were used to investigate the role of *SaMT2* in Cd and Zn tolerance. The yeast transformation was conducted using LiAc/PEG/ssDNA methods, as described by Gietz and Schiestl [16]. To obtain cells for transformation: Inoculate a single colony of the yeast strain with a sterile inoculation loop from a fresh SD (synthetic medium plus dextrose, 0.67% yeast nitrogen base, 2% D-glucose, and amino acids) plate into 5 ml of YPD medium (2% peptone, 1% yeast extracts, 2% D-glucose) and incubate overnight at 30°C. Add 2.5×10^8^ cells to 50 ml of YPD medium in a culture flask and incubate until the cell titer is at least 2×10^7^ cells ml^−1^. Cells were harvested by centrifugation at 3,000 g for 5 min and washed twice with sterilized water. Then the cells were re-suspended in 1.0 ml of sterile deionized water and pelleted by centrifugation (13 000×g for 30 sec). The supernatant was discarded and the transformation mixture {containing 240 µl PEG 3350 (50% w/v), 36 µl 1.0 M lithium acetate, 10 µl single-stranded carrier DNA (10 mg ml^−1^) and plasmid DNA (0.5–1 µg), and sufficient sterile deionized water to provide a final volume of 360 µl} were layered over the pellet. The mixture was vortex vigorously for 1 min and subjected to a heat shock at 42°C for 40 min. The transformation mixture was then centrifuged at 13 000×g for 30 sec to pellet the cells. After the supernatant was decanted, the cells were resuspended in 1.0 ml of sterile deionized water. Aliquots of the resuspended cells were plated onto SD-URA media. Plates were incubated for 2–3 days at 30°C until transformants were observed. Single colonies were picked from each transformant plate and established on fresh SD-URA plates.

For the metal tolerance assay, single colonies from SD-URA plates were cultured in liquid SD-URA medium until OD_600_ reached 1.0. After serial dilutions (OD_600_ = 0.1, 0.01, 0.001, 0.0001, respectively) were prepared, each dilution was spotted onto SD-URA medium with or without 5 mM ZnSO_4_ or 30 µM CdCl_2_. Plates were photographed after incubation at 30°C for 3 d.

For determination of metal concentration in yeast, transformants were grown in liquid SD-URA medium overnight. Then, cells were adjusted to OD_600_ = 0.2 in the presence of 10 µM CdCl_2_ or 100 µM ZnSO_4_ for Zn determination. After incubation for 48 h, the cells were harvested and washed with distilled water, 20 mM Na_2_EDTA and distilled water, respectively. Dry weight was determined after 3 days at 60°C. Cells were digested using 5 ml concentrated HNO_3_, incubated at 95°C for 2 h. The Zn and Cd concentrations were determined by using ICP-MS (Inductively Coupled Plasma Mass Spectrometry, Agilent 7500a, CA, USA).

### Heterogeneous expression of *SaMT2* in tobacco

The transformation of tobacco was constructed using the leaf disk method according to Horsch *et al* [17]. Surface-sterilized T_1_ seeds of two transgenic tobacco lines were germinated on Murashige Skoog (MS) plates containing 40 mg/L hygromycin to select hygromycin-resistance seedlings. For Zn/Cd tolerance analysis, the wild type (WT) and transgenic plants were transferred to MS plates containing 100 µM CdCl_2_ or 200 µM ZnSO_4_ for 14 d. To determine the Zn/Cd concentrations in plants, both WT and transgenic plants were transferred to hydroponic culture. One-month old plants were then treated with 50 µM CdCl_2_ or 100 µM ZnSO_4_ for one week. Cadmium and zinc concentrations in plant tissues were measured using ICP-MS as described by Yang *et al.* [13].

### Determination of SOD, POD, CAT and H_2_O_2_

For antioxidant enzyme activity determination, a 0.5-g aliquot of plant sample was homogenized in 5 ml potassium phosphate buffer (50 mM, pH 7.8). The homogenates were then centrifuged at 12000×g for 20 min at 4 °C. The supernatants were used for the analysis of enzyme activity. Superoxide dismutase (SOD) activity was determined by the photochemical method described by Giannopotitis and Ries [18]. One unit of the enzyme activity was defined as the amount of enzyme required to result in a 50% inhibition of the rate of nitro blue tetrazolium reduction measured at 560 nm. Catalase (CAT) activity was estimated according to Cakmak *et al.* [19]. The reaction mixture in a total volume of 2 ml contained 25 mM sodium phosphate buffer (pH 7.0), 10 mM H_2_O_2_. The reaction was initiated by the addition of 100 µl of enzyme extract and activity was determined by measuring the initial rate of disappearance of H_2_O_2_ at 240 nm (E = 39.4 mM^−1 ^cm^−1^) for 30 s. Peroxidase (POD) activity was measured as the increase of absorbance due to guaiacol oxidation [20]. The reaction mixture contained 25 mM phosphate buffer (pH 7.0), 10 mM H_2_O_2_, 0.05% guaiacol and 100 µl of enzyme extract. The reaction was initiated by the addition of H_2_O_2_. The oxidation of guaiacol was measured at 470 nm (E = 26.6 mM^−1 ^cm^−1^).

H_2_O_2_ was determined according to Loreto & Velikova [21]. Leaf tissues (0.07 g) were homogenized in an ice bath with 5 ml of 0.1% (w/v) trichloroacetic acid (TCA). The homogenate was centrifuged at 12,000×g for 15 min and 0.5 ml of the supernatant was added to 0.5 ml of 10 mM potassium phosphate buffer (pH 7.0) and 1 ml of 1 M KI. The absorbance of the supernatant was measured at 390 nm. The content of H_2_O_2_ was calculated by comparison with a standard calibration curve previously made by using different concentrations of H_2_O_2_.

### Statistical analysis of data

All data were statistically analyzed by using the SPSS package (version 20.0). All values were performed as means of three replicates. Data was tested at significant levels of p<0.05 using one-way ANOVA (analysis of variance). The graphical works were made by using Origin software.

## Results

### Clone and sequence analysis of *SaMT2*

A cDNA fragment of metallothionein like gene was obtained from RNA-seq of *S. alfredii*. RT-PCR and RACE techniques were used to obtain the full length cDNA of this gene, whose sequence was identified by BLAST search (www.ncbi.nlm.nih.gov/BLAST). The obtained cDNA encoded a 79 amino acids protein, which showed certain similarity to the cDNA of *AtMT2a* or *AtMT2b*. According to the amino acid sequences, it belonged to the Type 2 MTs, and was named *SaMT2* (GeneBank accession number: KJ862538).

**Figure 1:**
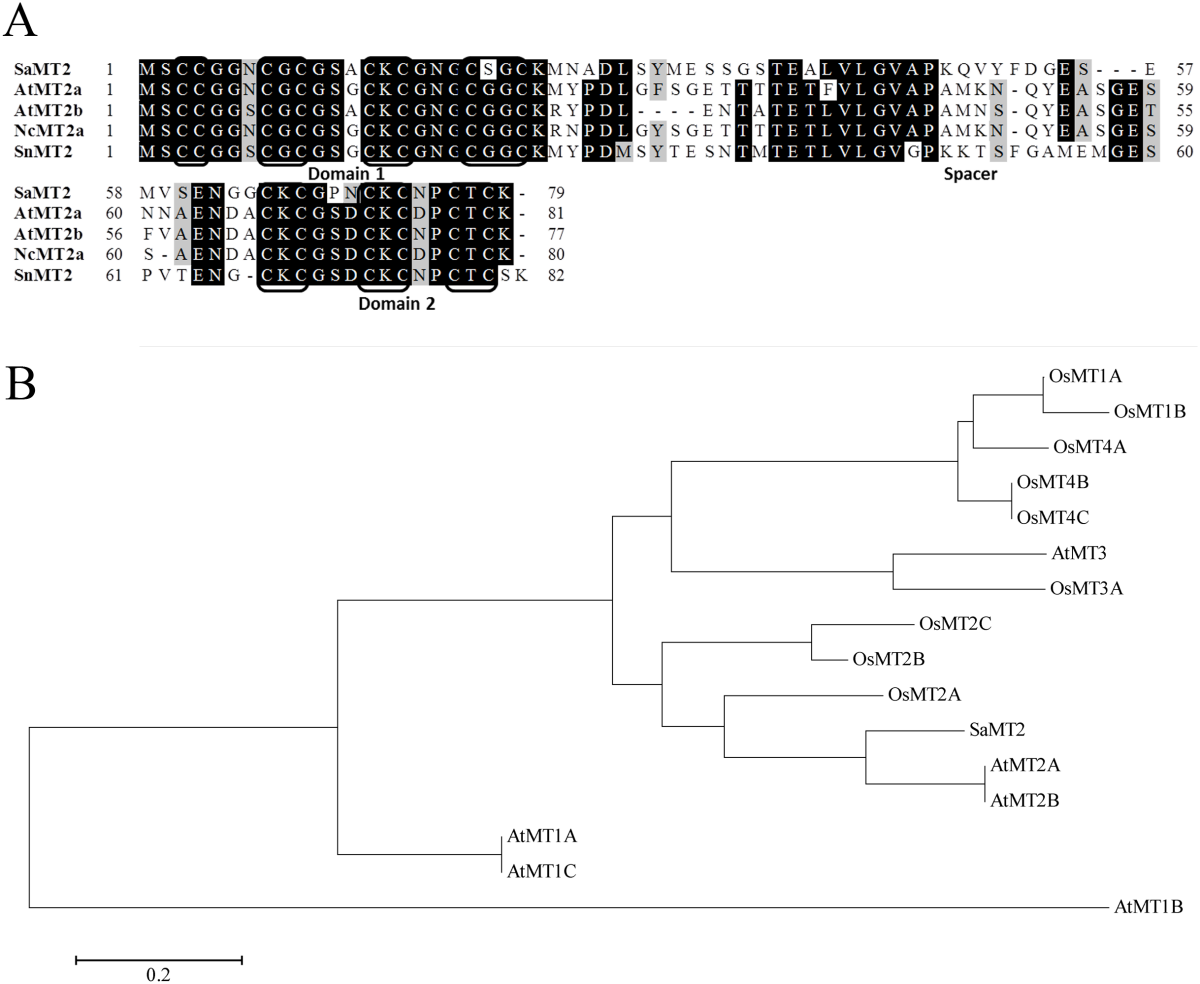
Sequence alignment and phylogenic analysis of *SaMT2* with other MTs. (A) The deduced amino acid sequences encoded by *SaMT2* were aligned with MTs from *Arabidopsis thaliana*, *Noccaea caerulescens* and *Solanum nigrum*. The cysteine-rich domains are boxed. (B) The phylogenic tree of *SaMT2* and MTs from Arabidopsis and rice.

Multiple sequence alignment of the deduced amino acid sequences of *SaMT2* with *AtMT2a* (*Arabidopsis thaliana*, NP_187550.1), *AtMT2b* (NP_195858.1), *NcMT2a* (*Noccaea caerulescens*, ACR46966.1) and *SnMT2* (*Solanum nigrum*, ACF10396.1) were conducted (Figure 1A). *SaMT2* shared 62%, 59%, 59% and 66% similarities with *AtMT2a*, *AtMT2b*, *NcMT2a* and *SnMT2*, respectively. Similar to other plant MT proteins, *SaMT2* contained two cysteine-rich domain separated by a large cysteine-free domain [22]. The cysteine-rich domains in the N-terminal region is CCxxxCGCxxxCKCxxxCxGC, which was highly conserved, and that in the C-terminal region contained three CxC motifs. The spacer region between the two terminal regions contained approximately 40 amino acids.

The phylogenetic tree of *SaMT2* and MTs from Arabidopsis and rice was conducted using MEGA software (Figure 1B). These MTs were divided into several groups and *SaMT2* was closely clustered with *AtMT2a* and *AtMT2b*.

### Expression analysis of *SaMT2* in *Sedum alfredii* Hance

**Figure 2:**
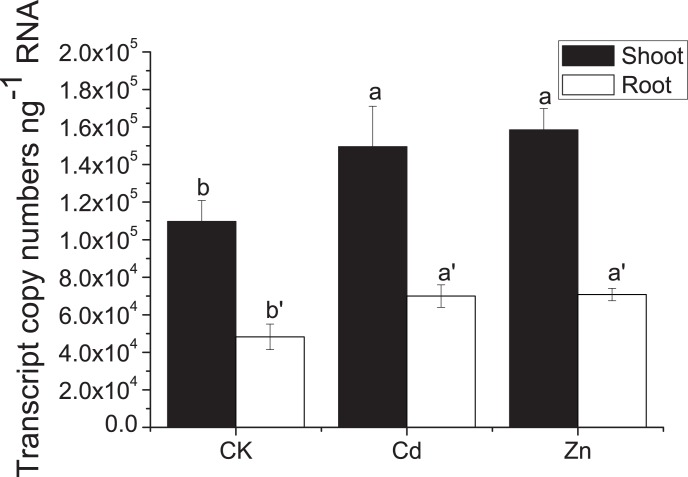
The expression level of *SaMT2* in *Sedum alfredii*. The transcript level of *SaMT2* induced by Cd and Zn treatments. The different letters above the columns indicate the significant difference between the treatments (p<0.05, Tukey’s test). CK represents the control group.

The expression of *SaMT2* was investigated using absolute quantitative RT-PCR. To investigate whether Cd or Zn were involved in the regulation of *SaMT2*, *S. alfredii* seedlings were treated with 100 µM CdCl_2_ or 500 µM ZnSO_4_ and were subjected to determine the transcript level of *SaMT2*. The expression level of *SaMT2* in roots was higher than that in shoots. The expression of *SaMT2* was significantly (p<0.05) increased in both roots and shoots treated with Cd and Zn (Figure 2).

### *SaMT2* enhanced cadmium but not zinc tolerance in yeast mutants

**Figure 3:**
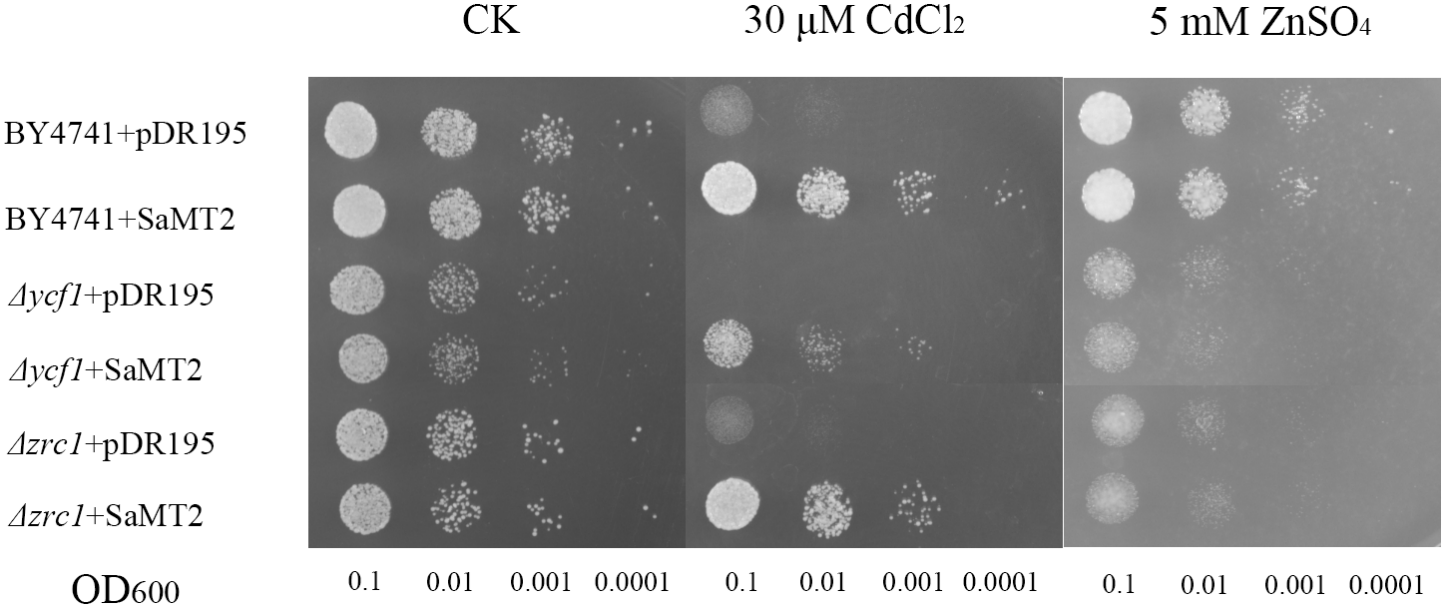
Cd and Zn tolerance of yeast cells expressing *SaMT2*. The *Saccharomyces cerevisiae* BY4741, *Δycf1* and *Δzrc1* yeast cells harboring pDR195 (vector control) or pDR195-*SaMT2* were grown in liquid SD selective medium. Cultures were adjusted to OD_600nm_ of 0.1 and serially 10-fold diluted in water. 10 µl aliquots of each dilution were spotted either on SD selective plates or on plates with 30 µM CdCl_2_ or 5 mM ZnSO_4_. After 3 days of incubation at 30°C, plates were photographed. CK represents the control group.

The *Δycf1* and *Δzrc1* yeast mutants and the wild type strain BY4741 were used to test the Cd and Zn tolerance ability of *SaMT2*. When grown in the control medium, yeasts containing either pDR195 or pDR195-*SaMT2* could grow well. When grown in a medium containing 30 µM CdCl_2_, the growth of yeast contain pDR195 was significantly inhibited; however, the expression of *SaMT2* could markedly mitigate this growth defect (Figure 3). However, the growth of yeasts in a Zn containing medium was not affected whether *SaMT2* was expressed or not (Figure 3).

**Figure 4:**
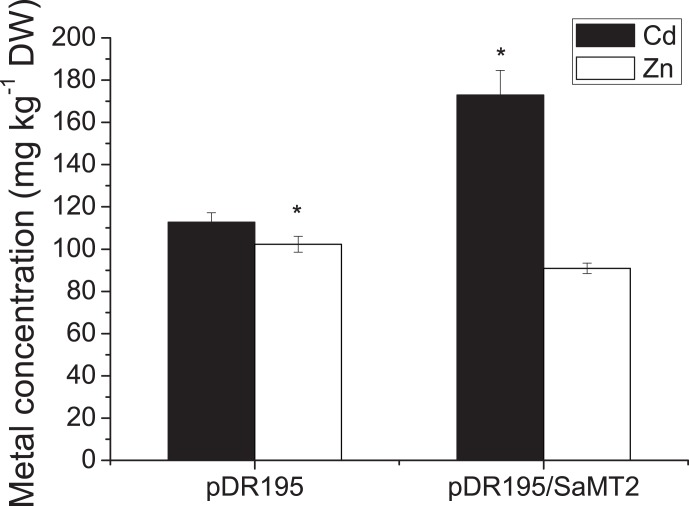
Cd and Zn concentration in *Δycf1* and *Δzrc1* yeast cells expressing *SaMT2*. The yeast transformants containing pDR195 or pDR195-*SaMT2* were grown in liquid SD selective medium with 30 µM CdCl_2_ and 100 µM ZnSO_4_ for *Δycf1 and* *Δzrc1*, respectively. Cells were incubated at 30°C for 48 h and metal contents were measured by ICP-MS. Results are averages (±S.E.) from three independent experiments done with four different colonies. The ‘*’ symbol indicates the mean values were significantly different at p<0.05 (Tukey’s test).

Similar trends were found for Cd and Zn concentrations in yeast mutants. Expression of *SaMT2* significantly increased the Cd concentration in yeast *Δycf1* mutant; however, it decreased the concentration of Zn in yeast *Δzrc1* mutant significantly (p<0.05, Figure 4).

### Overexpression of *SaMT2* in tobacco enhanced Cd and Zn tolerance and accumulation

To evaluate the functions of *SaMT2* in plants, transgenic tobacco plants were generated, ectopically expressing *SaMT2* under the control of CaMV 35S promoter. Three independent transgenic tobacco lines over-expressing *SaMT2* were selected for Cd and Zn tolerance analysis. The wild plants were used as control.

**Figure 5:**
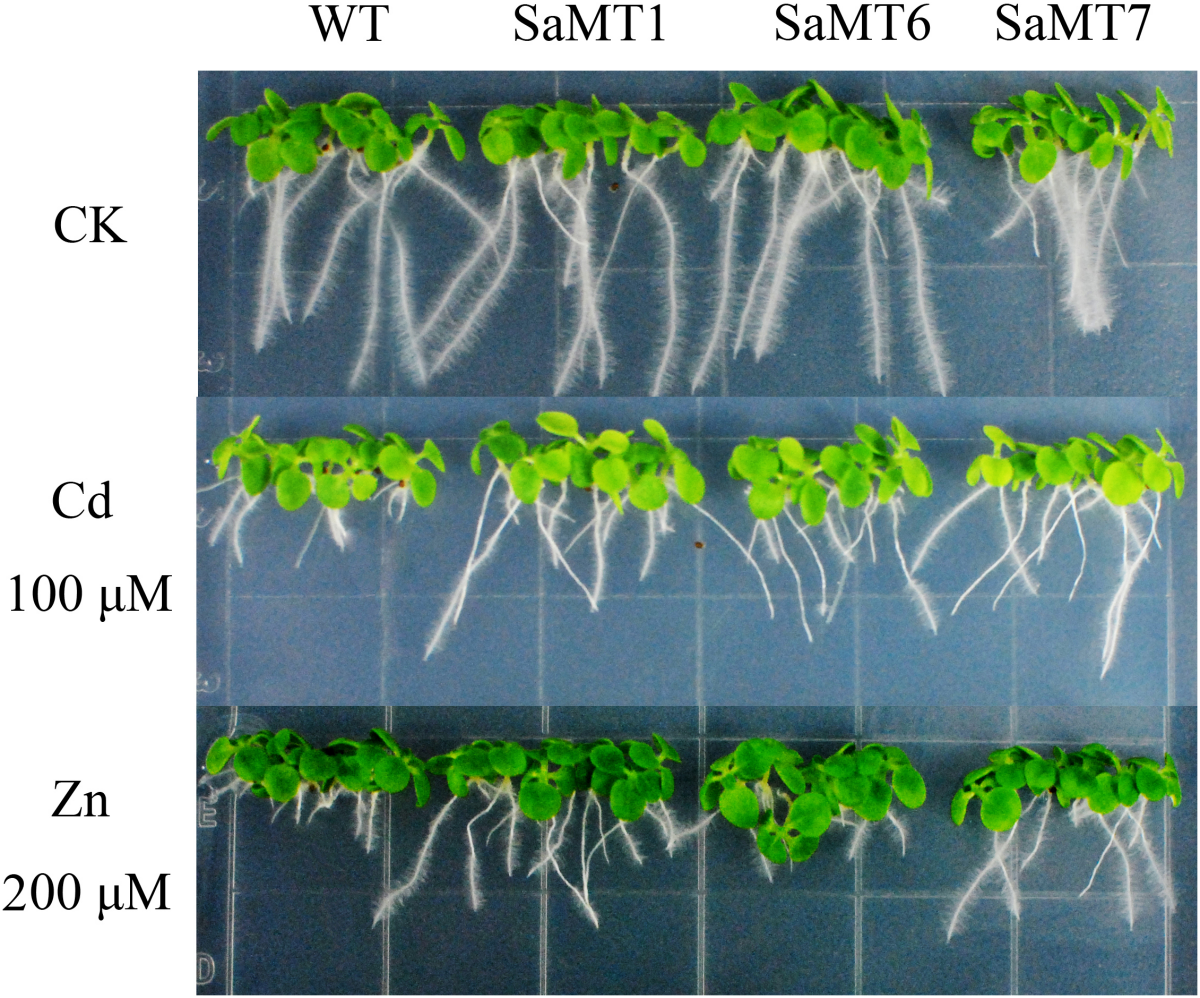
Metal tolerance analysis of transgenic tobacco plants over-expressing *SaMT2*. The figure shows the effect of 200 µM ZnSO_4_ or 100 µM CdCl_2_ on the growth of WT and transgenic plants on B5 medium. CK represents the control group.

**Figure 6:**
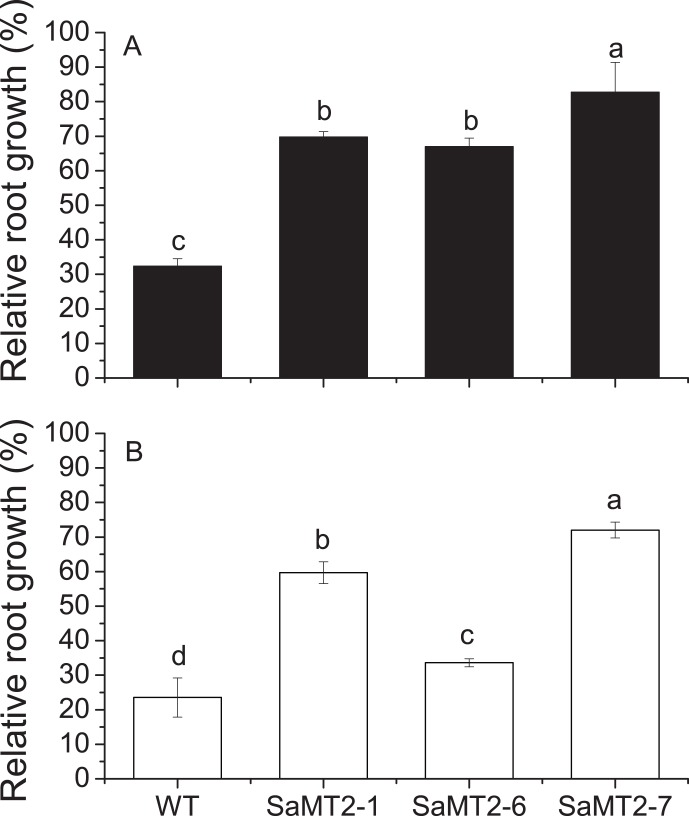
Relative root growth of transgenic tobacco plants. The relative root growth of WT and transgenic tobacco plants under Cd (A) and Zn (B) treatments. Different letters above the columns indicate a significant difference among different plant lines (p<0.05, Tukey’s test).

There was no difference in growth between wild type and transgenic plants under control condition. Exposure of the plants to 100 µM CdCl_2_ or 200 µM ZnSO_4_ significantly decreased root elongation and plant growth of both wild type and transgenic plants; however, the growth deficiency was less pronounced in transgenic plants (Figure 5). Under 100 µM CdCl_2_ or 200 µM ZnSO_4_ treatments, the root growth of wild type plants was decreased by 68% or 76%, compared to the control, respectively. However, the transgenic plants showed a significantly higher resistance to Cd and Zn. Compared to the control, the root growth of the transgenic plants was only decreased by 17%–33% under Cd treatment, and decreased by 28%–66% under Zn treatment (Figure 6).

**Figure 7:**
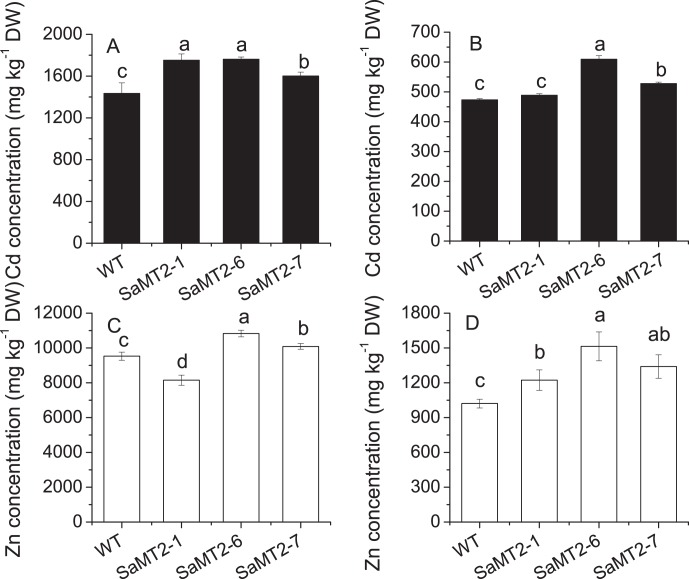
Cd and Zn concentrations in wild type amd transgenic tobacco lines overexpressing *SaMT2*. Three independent *SaMT2* over-expressing lines and wild-type tobacco were grown in nutrient solution containing 50 µM CdCl_2_, 100 µM ZnSO_4_ for 1 week. A: Cd concentration in roots, B: Cd concentrantion in shoots, C: Zn concentration in roots, D: Zn concentration in shoots. Results are means ± S.E. (n = 3). Different letter indicate the mean values were significantly different from WT tobacco determined by Tukey’s test (p<0.05).

Over-expression of *SaMT2* gene significantly increased both Cd and Zn concentration in transgenic tobacco plants (p<0.05) (Figure 7). Compared to the wild type plants, the Cd concentration was increased by 11–22% in the roots and by 3–28% in the shoots of the transgenic plants, respectively (Figure 7A, B). Except for the SaMT2-1 line, the Zn concentration was increased by 6–14% in roots and 20–48% in shoots of transgenic plants, respectively (Figure 7C, D). The SaMT2-6 line accumulated the highest amount of Cd and Zn among the three transgenic lines.

Based on the tolerance and accumulation of Cd and Zn in transgenic tobacco lines, the SaMT2-7 line - having similar Cd and Zn tolerance and accumulation level- was selected, to evaluate the reason of elevated tolerance to Cd and Zn of the transgenic plants. The plants treated with different metals were used to determine the activities of SOD, POD, CAT and the content of H_2_O_2_. The transgenic plant accumulated significantly less H_2_O_2_ in both roots and shoots than the WT plants under Cd and Zn treatments. The activities of SOD and POD were significantly increased in both roots and shoots of transgenic plants compared to that of wild type plants under Cd and Zn treatments (Table 1). For CAT activity, however, no significant difference was observed between WT and transgenic plants.

**Table 1:** The activities of SOD, POD CAT and the content of H_2_O_2_ in the shoot and roots of wild-type and transgenic tobacco plants.

	Metal treatment	SOD (U mg^−1^ Protein)		POD (nanokatals mg^−1^ Protein)		CAT (nanokatals mg^−1^ Protein)		H_2_O_2_ (µg g^−1^ FW)	
		WT	Transgenic	WT	Transgenic	WT	Transgenic	WT	Transgenic
Shoot	CK	20.1±0.4d	21.4±1.5d	20190.7±205.0d	20045.6±175.0d	36.7±5.0b	35.0±3.3b	154.4±10.0d	156.1±12.1d
	50 µM Cd	23.5±0.3c	28.6±1.2a	29267.5±720.1b	33821.7±688.4a	28.3±3.3c	45.0±1.7a	411.2±20.7a	167.9±10.1d
	100 µM Zn	24.3±0.5c	25.5±0.4b	26777.0±855.1c	30396.0±373.4b	25.0±1.7c	35.0±3.3b	267.3±9.7b	215.2±2.0c
Root	CK	24.2±0.4e	25.7±1.3e	30199.3±338.4e	31156.2±1171.9e	5.0±1.7b	3.3±1.7b	131.6±6.4d	132.0±7.4d
	50 µM Cd	30.2±0.6c	41.6±1.4a	46077.5±606.7b	54069.1±825.1a	5.0±1.7b	11.7±1.7a	400.5±23.0a	210.8±7.7c
	100 µM Zn	28.3±1.0d	34.2±0.7b	34723.6±270.0d	38681.0±1073.5c	6.7±1.7ab	10.0±1.7a	310.1±3.9b	198.6±10.0c

The data presented are mean ± SD of three replicates. Different letters indicate significant differences (p<0.05) among the different treatments and different plant lines.

## Discussion

Metallothioneins (MTs) are cysteine-rich proteins involved in metal tolerance of diverse living organisms. Plant metallothioneins can be divided into four subfamilies based on their sequence similarities and phylogenetic relationships [7], [8]. In the present study, the MT gene cloned from *S. alfredii* encoded a protein with two Cys-rich regions, showing high identity with the N- and C-terminal regions of type 2 MTs of other plants; therefore, this MT gene was named as *SaMT2*. *S. alfredii* is a Cd/Zn co-hyperaccumulator, which shows extremely high tolerance to Cd and Zn [12], [13]. Thus, It was hypothesized that *SaMT2* cloned from *S. alfredii* might be involved in Cd or Zn tolerance.

It has been reported that different MT genes have distinct tissue specific expression patterns in plants [8]. Generally, MT1s are predominantly expressed in roots, MT2s and MT3s in shoots [22], [23]. In the present study, *SaMT2* was more highly expressed in shoots of *S. alfredii* than in roots. Similar results have also been found in Arabidopsis, rice and other plants [22]–[25]. The expression of MT genes in plants is regulated by many factors, including metal ions, oxidative stress, and stresses such as heat, salt, wounding and so on [11]. Here, the expression of *SaMT2* was significantly increased in both roots and shoots of *S. alfredii* treated with Cd or Zn; in contrast, it has been reported that Cd and Zn do not induce the expression of *TcMT2* and *TcMT3* in *Thlaspi caerulescens* (now *Noccaea caerulescens*) another Cd/Zn hyperaccumulator [29].

The plant MTs are suggested to be involved in metal homeostasis or tolerance, such as Cu, Cd and Zn. When expressed in yeast or *E. coli*, the plant MTs are able to restore Cu, Cd and Zn tolerance [9], [11], [26], [27]. In the present study, Cd and Zn induced the expression of *SaMT2*, suggesting its possible involvement in Cd and Zn tolerance. This was confirmed in yeast and tobacco plant overexpressing *SaMT2*, which exhibited increased Cd and Zn tolerance and accumulation. Previous studies also reported enhanced tolerance and accumulation of Cd or other heavy metals by over-expressing plant MT genes. For example, the over-expression of *Cajanus cajan* MT1 enhances Cd and Cu tolerance in *E. coli* and Arabidopsis [27]. The expression of *Colocasia esculenta CeMT2b* increases Cd tolerance and accumulation in *E. coli* and tobacco [28]. However, Hassinen et al. [29] have observed that MT expression and Cd accumulation are not correlated among *T. caerulescens* accessions. Furthermore, the overexpression of *TcMT2* and *TcMT3* do not increase Cd accumulation in Arabidopsis shoots. On the other hand, Lv et al. [30] have observed that the ectopic expression of either *BcMT1* or *BcMT2* does increase Cd tolerance, but not the Cd accumulation in Arabidopsis shoots and roots. Thus, the MT genes may have different specific functions, depending on plant species.

The plant MTs are thought to function as metal chelators or ROS scavengers in heavy metal stress [8]. On one hand, plant MT proteins are supposed to have binding activities to heavy metals, such as Cd, Zn and Cu [7], [8]. In the present study, the ectopic expression of *SaMT2* in tobacco enhanced Cd tolerance and accumulation, which might be due to reduced activities of free Cd ions in the cytoplasm, by the binding of overexpressed MT protein and Cd. On the other hand, MTs can also function as ROS scavengers which can reduce the ROS induced by Cd or other metals [8]. The plants exposure to heavy metals, such as Cd, can produce ROS and oxidative stress. The present study demonstrated that overexpression of *SaMT2* could significantly reduce H_2_O_2_ in tobacco exposure to excess Cd. Several studies have demonstrated that MTs can effectively scavenge ROS in plants. Over-expression of *BcMT1*, *BcMT2* [30], *EhMT1* [31], *pCeMT* [9] reduces ROS production in transgenic plants. Using recombinant GhMT3a protein, Xue *et al.* [32] have demonstrated that GhMT3a can scavenge ROS *in vitro*. Plants themselves have developed various antioxidant defense mechanisms to protect from deleterious effects of ROS. One of them is the enzymatic system, which includes SOD, APX, POD, and CAT. Plants overexpressing MT genes show higher antioxidant enzyme activities [8]. The present study demonstrated that tobacco plants overexpressing *SaMT2* showed higher SOD and POD activities than wild type plants, indicating that *SaMT2* might also act as an activator of antioxidant enzyme system.

It has been demonstrated that MTs are not related with Cd or Zn tolerance and accumulation in hyperaccumulator *T. caerulescens*, even though the expression of MT genes varies among *T. caerulescens* accessions [29]. However, in the present study, ectopic expression study in yeast and tobacco revealed that *SaMT2* might play certain roles in Cd and Zn tolerance and accumulation. It is not certain whether *SaMT2* is directly involved in Cd or Zn tolerance in *Sedum alfredii*. There are clear evidence that MTs are not directly related in Zn or Cd tolerance in *T. caerulescens* [29]. Data from the present study demonstrated that *SaMT2* might be involved in the Cd or Zn induced antioxidant stress in *Sedum alfredii*. However, the exact role of *SaMT2* in metal tolerance and accumulation in *Sedum alfredii* needs to be examined by further study in the future.

In conclusion, *SaMT2* is a metallothionein gene cloned from Cd/Zn hyperaccumulator *Sedum alfredii* Hance. Overexpression of this gene could significantly enhance Cd tolerance and accumulation in yeasts and tobacco plants. The mechanism of the elevated Cd tolerance and accumulation by overexpressing of *SaMT2* includes binding of *SaMT2* with Cd and improving the antioxidant system.
